# Complete Genome Sequence of a *Gluconacetobacter hansenii* ATCC 23769 Isolate, AY201, Producer of Bacterial Cellulose and Important Model Organism for the Study of Cellulose Biosynthesis

**DOI:** 10.1128/genomeA.00808-16

**Published:** 2016-08-11

**Authors:** Sarah Pfeffer, Kalpa Mehta, R. Malcolm Brown

**Affiliations:** University of Texas at Austin, Molecular Biosciences, College of Natural Sciences, Austin, Texas, USA

## Abstract

The cellulose producer and model organism used for the study of cellulose biosynthesis, *Gluconacetobacter hansenii* AY201, is a variant of *G. hansenii* ATCC 23769. We report here the complete nucleotide sequence of *G. hansenii* AY201, information which may be utilized to further the research into understanding the genes necessary for cellulose biosynthesis.

## GENOME ANNOUNCEMENT

The genus *Gluconacetobacter* contains several strains of Gram-negative bacteria that are particularly efficient producers of pure, highly crystalline cellulose, one of which is *Gluconacetobacter hansenii*, formerly known as *Acetobacter xylinum*. The export of this crystalline cellulose into the culture medium results in a membrane located at the air-liquid interface called bacterial cellulose (BC) (1–3). Because of its distinctive properties, BC is particularly well suited for medical, industrial, and commercial applications due to its ultrafine reticulated structure, high crystallinity, great mechanical strength, high water-holding capacity, moldability during formation, and biocompatibility (4–7). The results presented in this report will provide insight into the molecular mechanisms of bacterial cellulose biosynthesis and add to the study of the *Gluconacetobacter* genus.

*G. hansenii* AY201 (ATCC 23769) was developed from an isolate of *G. hansenii* ATCC 23796 that exhibited non-wild-type pellicle and colony morphology (unpublished data). It is an important model organism for the study of cellulose biosynthesis (8–10). However, until now, its genome had not been available for study. The DNA of *G. hansenii* AY201 was extracted and subjected to sequencing using an Illumina HiSeq 2000 PE100 system (University of Texas at Austin, ICMB Core Facility). The reads were assembled into contigs using Velvet version 1/2/02 (11) and downloaded into Geneious version 8.1.2, which revealed that it is approximately 3.35 Mbp in size with a GC content of 55.9% (12); a total of 6,443 open reading frames were predicted using Glimmer (13). Preliminary annotation data on contigs containing cellulose synthase genes were determined. The complete annotation of the full genome is in progress.

A homology comparison to *G. hansenii* ATCC 23769 (GenBank accession no. AB091060) was performed and resulted in a 95% identity to *G. hansenii* AY201. Previous studies have determined that *G. hansenii* AY201 contains at least two similar but nonidentical cellulose synthesizing regions, the *acsABCD* operon and the *acsAII* coding region (9, 10). Investigations into the genome of *G. hansenii* ATCC 23769 indicated that the organism contains a total of three separate coding regions for cellulose biosynthesis: *acsABCD*, *acsAII*, and *acsABC* (9). A homology comparison of the shared cellulose-synthesizing regions revealed a sequence identity of 100%. The *acsABCD* operon is flanked by genes coding for proteins which have been determined to be essential for proper cellulose biosynthesis to occur: *cmcAx, ccpAx,* and *bglAx* (14–17). The genes flanking the *acsABCD* operon also shared 100% sequence identity to *G. hansenii* ATCC 23769.

Since *G. hansenii* AY201 is a model organism for genetic study, further investigations into both cellulose synthase-coding regions and why an isolate of *G. hansenii* ATCC 23769 lost a third may aid in providing a better understanding of the mechanisms necessary for cellulose biosynthesis to occur.

### Accession number(s).

This whole-genome shotgun project has been deposited in DDBJ/ENA/GenBank under the accession number LUCI00000000. The version described in this paper is the first version, LUCI01000000.
